# Mid-childhood acne conglobata successfully treated with adalimumab

**DOI:** 10.1016/j.jdcr.2025.11.027

**Published:** 2025-12-04

**Authors:** Andrés Vidal González, Sergio López Alcázar, Rafael Escudero Tornero, Rocío Maseda Pedrero, Raúl De Lucas Laguna, Marta Feito Rodríguez

**Affiliations:** Dermatology, Hospital Universitario La Paz, Madrid, Spain

**Keywords:** acne conglobata, adalimumab, nodulocystic acne

## Introduction

Acne vulgaris is a common dermatologic condition affecting both pediatric and adolescent populations. Its clinical presentation, differential diagnosis, and potential association with systemic disease vary by age of onset. Based on age, acne is typically classified into distinct subgroups: neonatal acne (0-6 weeks), infantile acne (1 month-1 year), mid-childhood acne (1-7 years), preadolescent acne (7-12 years), and adolescent acne (12-18 years).^1^

Acne conglobata represents a severe, chronic, nodulocystic form of acne characterized by confluent inflammatory nodules and abscesses that may coalesce into large masses or plaques, often leading to scarring.^2^ The condition most commonly affects the trunk and, less frequently, the face. This disfiguring variant is rarely seen in children between 1 and 7 years of age.^3^

Although oral isotretinoin remains the gold standard for the management of acne conglobata, adjunctive therapies such as systemic or intralesional corticosteroids are often used. Biologic agents, including adalimumab, have only been reported in isolated, treatment-refractory cases in adults and adolescents.^4^

We report a rare case of mid-childhood acne conglobata successfully treated with adalimumab in a 2-year-old child given the failure of conventional agents.

## Case report

A 1-year-old boy was referred to the emergency department by his pediatrician for evaluation of facial lesions that had been present for 3 months. Prior treatments included multiple topical antibiotics (mupirocin and fusidic acid) and a course of oral amoxicillin, with minimal clinical improvement. The patient remained afebrile and exhibited no systemic symptoms.

Physical examination revealed a purulent, erythematous–violaceous nodule approximately 2 cm in diameter on the right temple, along with several papules and pustules scattered across both cheeks. Point-of-care ultrasonography demonstrated findings consistent with a cutaneous abscess. The blood count and biochemistry showed no abnormalities. A culture through aspiration was done from the purulent drainage, which proved to be sterile.

An initial differential diagnosis included acne conglobata versus granulomatous rosacea. There was no relevant family history, with no previous cases of acne, hidradenitis suppurativa, pilonidal sinus, or folliculitis decalvans. The lesion was incised and drained, followed by intralesional injection of betamethasone. Oral azithromycin (3.5 mL of 200 mg/5 mL suspension, administered 3 times weekly) was initiated.

**Fig 1:**
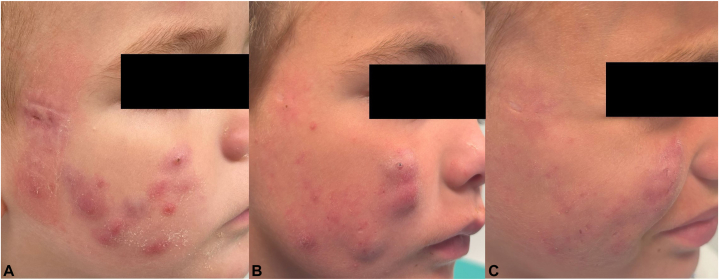
Evolution of lesions on the right cheek lesions: **(A)** confluent skin abscesses and inflammatory papules on the first visit; **(B)** skin abscesses, inflammatory papules and cribriform scarring at the start of adalimumab treatment; **(C)** clinical improvement of previous lesions after 3-month treatment with adalimumab.

Despite treatment, the patient experienced progressive clinical deterioration, with the development of new nodulocystic lesions and facial abscesses, raising strong suspicion for acne conglobata (Fig 1). Multiple therapeutic modalities were trialed, including oral isotretinoin (2.5 mg/day) for 8 months, oral ivermectin cycles, oral corticosteroid pulses, intralesional corticosteroid injections, and pulsed dye laser sessions, all with limited response.

**Fig 2:**
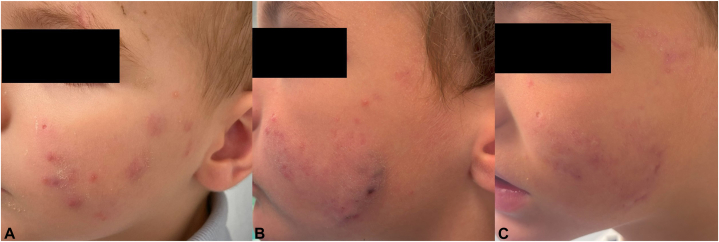
Evolution of lesions on the left cheek: **(A)** skin abscesses and inflammatory papules on the first visit; **(B)** skin abscesses, inflammatory papules and cribriform scarring at the start of adalimumab treatment; **(C)** clinical improvement of previous lesions after 3-month treatment with adalimumab.

Given the refractory nature of the disease, adalimumab 20 mg subcutaneously every 15 days was initiated. An interferon-γ release assay (IGRA) and hepatitis serology tests were performed prior to the initiation of anti-TNF therapy. Clinical improvement became noticeable after 2 doses, with a more marked and sustained response observed after approximately 3 months of treatment, with complete cessation of new lesions and gradual resolution of existing nodules (Fig 2).

The patient was also evaluated by Pediatric Endocrinology, where the clinical evaluation ruled out hyperandrogenism. Moreover, hormonal assessment was done with normal levels of thyroxine, thyroid stimulating hormone, testosterone, androstenedione, 17-hydroxyprogesterone, DHEA, and DHEA-S. The X-ray of the left hand and wrist showed normal bone age. The patient was referred to Immunology to rule out autoinflammatory diseases that can present with sterile skin abscesses such as PAPA, DIRA, DITRA, PASH, PAPASH, or CANDLE syndromes. Workup was unremarkable, excluding associated autoinflammatory conditions by performing a targeted NGS (next-generation sequencing) panel of 87 genes associated with autoinflammatory disease (which included *PSTPIP1, IL1RN, IL36RN, NCSTN*, *PSENEN*, *PSEN1, PSMB8*, *PSMB9* among others).^5^

## Discussion

Acne conglobata is a severe and chronic variant of acne vulgaris, typically affecting adolescents and young adults. It is characterized by inflammatory nodules, abscesses, sinus tracts, and scarring, primarily on the trunk and face. The occurrence of acne conglobata in mid-childhood (ages 1-7) is exceedingly rare, with very few cases described in the literature.^6^

Prepubertal acne, particularly in children under 7, warrants a thorough evaluation for potential underlying endocrine or systemic causes. Clinical guidelines recommend assessment of androgen levels, bone age, and signs of virilization to rule out hyperandrogenism and other hormonal disorders.^7^ In our case, hormonal studies and immunologic evaluation were unremarkable, supporting the diagnosis of idiopathic, severe acne conglobata.

Initial management of nodulocystic and conglobate acne typically involves systemic antibiotics, oral isotretinoin, and, in certain cases, adjunctive corticosteroids. However, a subset of patients remains refractory to these treatments. Biologic agents, particularly TNF-α inhibitors such as adalimumab, have shown promise in isolated reports of severe acne and acne fulminans in adolescents and young adults.^8^^,^^9^ Their anti-inflammatory effects can be beneficial in highly resistant forms of acne with inflammatory and scarring potential.

Our patient, a 2-year-old with treatment-refractory acne conglobata, demonstrated significant clinical improvement following the initiation of adalimumab. While this biologic agent is not approved for acne vulgaris, its safety and efficacy have been well-documented in pediatric populations for other inflammatory dermatoses, such as hidradenitis suppurativa and psoriasis^9^^,^^10^ Furthermore, pediatric data supports its tolerability and favorable safety profile even in children under 4 years old when used with appropriate monitoring.^11^

The early recognition of severe or atypical acne presentations in pediatric patients is crucial, not only to initiate prompt and appropriate therapy, but also to prevent long-term scarring and psychosocial consequences. Early dermatologic referrals are recommended for prepubertal children with nodulocystic or recalcitrant acne, especially when the disease is extensive or unresponsive to first-line treatments.^12^

In conclusion, this report presents the first documented case of mid-childhood acne conglobata successfully treated with adalimumab. It highlights the potential utility of TNF-α inhibitors in rare, severe, and treatment-resistant pediatric acne. While biologic therapy is not standard for acne management, this case supports its consideration in selecting pediatric patients following multidisciplinary evaluation. Further studies are warranted to better define its role in pediatric dermatology and to establish formal treatment guidelines for this unique patient population.

## Conclusion

This case highlights a rare presentation of acne conglobata in a mid-childhood patient, a clinical scenario seldom described in the literature. After failure of multiple conventional therapies, treatment with adalimumab resulted in rapid and sustained clinical improvement, with excellent tolerability. Although off-label, biologic therapy may represent a valuable alternative for managing severe, treatment-refractory acne in pediatric patients, particularly when standard options prove ineffective. Early recognition, multidisciplinary evaluation, and individualized therapeutic strategies are essential to optimize outcomes and prevent long-term complications in this vulnerable population.

## Conflicts of interest

None disclosed.
